# Aetiology and Outcome of Childhood Convulsive Status Epilepticus: A tertiary care experience in Oman

**DOI:** 10.18295/squmj.8.2024.050

**Published:** 2024-08-29

**Authors:** Areeba Wasim, Shihab S. Al Maawali, Abdulrahman S. AlJabri, Fatema Al Amrani, Faraz Ahmad, Ahmed Mansi, Amna Al Futaisi

**Affiliations:** 1 Department of Child Health, Sultan Qaboos University Hospital, University Medical City, Muscat, Oman; 2 College of Medicine and Health Sciences, Sultan Qaboos University, Muscat, Oman; 3 Department of Child Health, Sultan Qaboos University, Muscat, Oman

**Keywords:** Etiology, Outcome, Convulsive Status Epilepticus, Rankin Scale

## Abstract

**Objectives:**

This study aimed to evaluate the aetiology, management and outcomes of convulsive status epilepticus (CSE) in children and highlight the factors influencing patient outcomes in such cases.

**Methods:**

In a retrospective study spanning the 2020–2023 period, 93 children with CSE treated at Sultan Qaboos University Hospital’s emergency department (ED), high dependency unit (HDU) and intensive care unit (ICU) were analysed. The Modified Rankin Scale at discharge was used to determine CSE outcomes.

**Results:**

Among the 93 children studied (mean age 4.84 ± 3.64 years), predominantly Omani (92.47%), 14 aetiologies were noted. Of them, acute symptomatic (37.7%) and febrile status (31.2%) were the primary causes of CSE. Diazepam was administered as the first-line treatment in 58 (67.44%) cases, with a median seizure duration of 45 minutes. Successful seizure control was achieved in 71 (76.34%) cases within 60 minutes. A return to baseline was observed in 55.9% of cases, while mortality and disability were noted in 5.38% and 38.7% of cases, respectively. For 17 cases, aetiology and duration significantly impacted patient outcomes (*P* <0.05).

**Conclusion:**

Acute symptomatic status is the most common aetiology of CSE. A longer duration of CSE is associated with higher mortality and neurological disability. Prompt and appropriate management of CSE is essential. Furthermore, identifying and treating the underlying cause of CSE is a crucial step in reducing its duration and improving patient outcomes.

**Advances in Knowledge**

*- Among the 93 children, acute symptomatic aetiologies were considered the primary cause of convulsive status epilepticus (CSE)*.
*- Prolonged CSE duration correlated with increased mortality and neurological disability*.
*- First-line antiseizure medications, particularly benzodiazepines like Diazepam, were commonly administered regarding CSE treatment*.
*- Successful seizure control was achieved in 76.34% of studied patients within 60 minutes*.
*- Subsequently, 55.9% of patients returned to baseline, 5.38% died and 38.7% experienced disability*.

**Applications to Patient Care**

*- This study’s outcomes offer valuable recommendations regarding the enhancement of patient care practices related to the management of childhood CSE*.
*- By identifying acute symptomatic aetiologies as the most common cause of CSE and linking prolonged CSE duration to poorer outcomes, clinicians can prioritise early intervention and tailored CSE treatments*.
*- This study’s findings underscore the critical importance of swift and appropriate CSE management, emphasising the concomitant need for multidisciplinary collaboration among healthcare providers*.
*- Ultimately, these insights will facilitate the improvement of CSE patient care protocols, potentially reducing mortality rates and enhancing the quality of life of children affected by CSE*.

Convulsive status epilepticus (CSE) is the most common neurological emergency among children and has a diverse aetiology. If prolonged, it can result in unfavourable patient outcomes, particularly in cases of refractory and super-refractory status epilepticus.^1^ CSE is characterised by continuous seizures lasting longer than 5 minutes or by repetitive seizures without the patient regaining consciousness. This condition occurs when the mechanisms that normally terminate seizures fail, resulting in neuronal injury and devastating neurological consequences.^2^ The global estimated incidence of status epilepticus (SE) in childhood is 20 cases per 100,000 per year, with the highest incidence being observed among children under the age of 5 years. This fact underscores the need for increased vigilance in detecting seizures, especially in young children.^3^^,^^4^

The International League Against Epilepsy’s (ILAE) defines CSE with two operational dimensions: the length of seizure and the time point (t1) beyond which the seizure should be considered as ‘continuous seizure activity’ necessitating treatment, and the time point (t2), which refers to the duration of ongoing seizure activity after which there is a risk of long-term consequences. For convulsive SE, both time points—t1 at 5 minutes and t2 at 30 minutes—are crucial for interpreting the short- and long-term consequences of CSE.^5^ Moreover, it is estimated that 23–48% of children with CSE progress to refractory SE; which can lead to varying outcomes.^6^

CSE is typically managed using specific national or local treatment algorithms. First-line treatment for CSE is administered when a tonic-clonic or motor clonic seizure lasts for more than 5 minutes (impending or premonitory CSE), while second-line treatment is given if CSE persists after two doses of first-line treatment (established CSE).^7^ Robust randomised clinical trial (RCT) evidence supports the use of benzodiazepines as first-line treatment, with phenytoin, levetiracetam and sodium valproate serving as second-line treatments.^8^

Aetiology is the most critical factor in determining the outcomes of CSE, although it cannot always be easily defined in emergency settings. The causes of CSE range from febrile and acute symptomatic conditions such as stroke, CNS infections, intoxication and metabolic disorders to remote symptomatic conditions like post-stroke, post-meningitic, postencephalitic, progressive encephalopathies such as electroclinical syndromes, neurodegenerative disorders and CNS tumours or even cryptogenic/unknown conditions.^9^ Febrile illnesses are considered the most common cause of CSE in children, accounting for approximately 33–35% of all cases. Some children with genetic epilepsy—specifically Dravet syndrome and PCDH19—may also experience febrile CSE, as these syndromes usually manifest between 3–12 months of age in the form of ‘febrile seizures’.^10^ Meanwhile, acute symptomatic aetiology with impaired consciousness at the outset is frequently associated with high morbidity and increased costs, particularly in cases of refractory disease, necessitating detailed and exhaustive workups.^11^

The available literature on this topic suggests that CSE is significantly linked to morbidity. However, there is insufficient data to determine whether severe outcomes are solely the results of CSE or influenced by factors such as patient’s age, management guidelines and timing of arrival at healthcare settings. To swiftly manage and prevent the refractoriness of CSE, which is associated with high-cost profiles and significant morbidity, this study aimed to identify the causes, management practices and outcomes of children with CSE. It sought to provide evidence that would enable clinicians to assess the early outcomes of CSE patients while considering the impact of predictive factors that significantly influence the prognosis of CSE.

## Methods

This retrospective cohort study was conducted at Sultan Qaboos University Hospital (SQUH), Muscat, Oman. It involved children affected by CSE who were admitted in the emergency department (ED), high dependency unit (HDU) and paediatric intensive care unit (ICU) between June 2020 and June 2023. Data were collected using electronic patient records through the TrakCare® system (Intersystems, Cambridge, Massachusetts, USA) available in SQUH. A non-probability consecutive sampling technique was employed in this study.

The inclusion criteria for the study encompassed patients of either gender, aged between 1 month and 12 years, who presented with CSE, regardless of seizure type or cause. Patients with nonconvulsive SE were excluded due to the difficulty in clinically interpreting their seizure duration and response to antiseizure medication. Additionally, patients with alternative diagnoses resembling CSE (e.g., those affected by status dystonicus and psychogenic non-epileptic attacks) were also excluded. For this study, CSE was operationally defined as continuous seizure activity lasting for more than 5 minutes and requiring antiseizure treatment for abortion, in accordance with the ILAE definition.^12^

Considering a specific timeframe, this study included 93 patients who met the operational definition of a CSE-affected patient. It documented the patients’ demographic information, including age, gender, nationality, seizure type (according to ILAE classification), seizure duration and the cause of CSE. In cases where convulsive SE occurred in a previously normal child within 1 week of acute CNS injury (such as bacterial, viral or autoimmune encephalitis), acute demyelinating disorders or acute stroke, it was labelled as an acute symptomatic aetiology.^13^ Furthermore, a child (aged 6 months to 5 years) reporting a prolonged febrile seizure was defined as a CSE patient based on an episode of fever without CNS infection.^14^

Remote symptomatic aetiology is defined as a type of epilepsy caused by a known risk factor, such as a previous stroke or head injury, occurring at least a week after the CNS insult.^15^ Furthermore, progressive encephalopathy encompasses neurodegenerative, epileptic and neurometabolic disorders, while static encephalopathy includes cerebral palsy. Idiopathic epilepsy is characterised by a second unprovoked seizure (leading to SE) and remains unclassified when it cannot be placed into other categories.^16^

In this context, the present study reviewed various tests—including neuroimaging, cerebrospinal fluid analysis (if needed), electroencephalography (EEG) and other relevant tests—to determine the cause of CSE in the selected children. It recorded the medical history, previous test results and treatment details of patients who had experienced a previous neurological insult, as well as the management practices related to their CSE. Patient outcomes were assessed using a modified Rankin score (0–6). These outcomes were classified into 3 categories based on this score: return to baseline (score 0–1), neurological disability (score 2–5) or mortality (score 6). Furthermore, their neurological disability was categorised as mild (score 2), moderate (score 3) and severe (scores 4–5).^17^–^18^ To determine the outcomes of previously neurologically disabled children, this study also considered their baseline score on the Glasgow Coma Scale along with the modified Rankin score.

The collected data were analysed using SPSS, Version 25 (IBM Corporation, Armonk, New York, USA). Numeric variables, such as patient age, seizure duration and time interval to control seizures, were expressed as mean ± standard deviation. Categorical variables, including gender, demography, seizure type, management options, aetiology of CSE and patient outcomes, were presented as frequencies and percentages. The association between patient outcomes and different variables—such as aetiology, duration of SE and the relationship between aetiology, seizure type and age—was determined using the Chi-squared test. A *P*-value ≤0.05 was considered statistically significant.

Ethical approval for this study was obtained from the hospital institution’s research ethical committee.

## Results

This study included 93 children—52 (55.9%) males and 41 (44.1%) females—with a male-to-female ratio of 1.3:1 [Table 1]. The patients’ average age was 4.84 ± 3.64 years. Most patients (86 patients, constituting 92.47% of the sample) were ethnic Omanis, while 7 (7.53%) patients were of non-Omani origin. The age distribution revealed that 41 (44.1%) patients were aged between 1 month to 2 years, 35 (37.6%) patients were aged between 2 to 6 years and 17 (18.3%) patients were aged between 6 to 12 years. Generalised seizures were the most common seizure type, affecting 49 patients (52.7%), followed by focal seizures (n = 17; 18.3%) and focal to bilateral tonic-clonic seizures (n = 24; 25.8%) [Table 1]. A mixed seizure type (comprising tonic, clonic and myoclonic seizures) was reportedly in 3 (3.2%) patients, mostly those with either static or progressive encephalopathy. Generalised seizures were significantly associated with aetiology (*P* ≤0.01) and were most commonly linked to an acute aetiology. Acute symptomatic aetiology was the most common cause in 35 (37.6%) patients, followed by febrile status in 29 (31.2%) patients, remote symptomatic aetiology in 4 (4.3%) patients, progressive encephalopathy in 11 (11.8%) patients, static encephalopathy in 4 (4.3%) patients, idiopathic epilepsy in 9 (9.7%) patients and unclassified aetiology in only 1 patient. Acute symptomatic aetiology and febrile status were noted in the early age groups, whereas progressive encephalopathy was commonly seen in late childhood (*P* ≤0.01).

In total, 86 (92.47%) patients received first-line interventions, while 63 (67.74%) required second-line antiseizure medications. Another 36 (38.7%) patients needed third-line medication for seizure control. The most commonly used antiseizure medications for CSE management were diazepam, phenytoin and levetiracetam [Table 2]. A significant number of patients received benzodiazepines as first-line measures, including diazepam (67.44%) and midazolam (4.65%), aimed at stopping their seizures. Phenytoin (42.86%) and levetiracetam (22.22%) were among the most commonly used second-line antiseizure drugs. Furthermore, midazolam infusion (30.56%) and phenytoin (27.78%) were the most commonly used third-line antiseizure drugs in this study. Out of 86 patients, 23 (26.74%) responded to the first-line treatment; out of the remaining 63 patients, 27 (42.86%) responded to the second-line treatment. The median time to control the seizures was 45 minutes, with a range of 30–600 minutes. Notably, this study showed that 71 (76.34%) seizure cases were successfully controlled within 60 minutes. The prolonged duration of seizures (exceeding 6 hours) was mainly noted in aetiologies like progressive encephalopathy and remote symptomatic seizures. The duration of seizure control had a strong association with the aetiology of SE (*P* = 0.027) [Table 3].

Out of the 52 patients who recovered (returned to baseline), 41 (78.85%) experienced controlled seizures within 60 minutes, while 11 (21.15%) had uncontrolled seizures lasting over 60 minutes [Table 4].

The patients who received diazepam had a mean age of 4.54 years (range: 0.17–12 years), those who were administered phenytoin had a mean age of 4.71 years (range: 0.17–12 years) and those who received levetiracetam had a mean age of 3.95 years (range: 0.42–12 years).

Of the total sample, 91 patients were admitted in the hospital. The mean hospital stay duration was 5.82 days (range: 1–55 days). Among the 91 admitted patients, 54 (58.89%) stayed in the hospital for over 2 days, while 37 (41.11%) stayed for 2 days or less.

**Figure 1:**
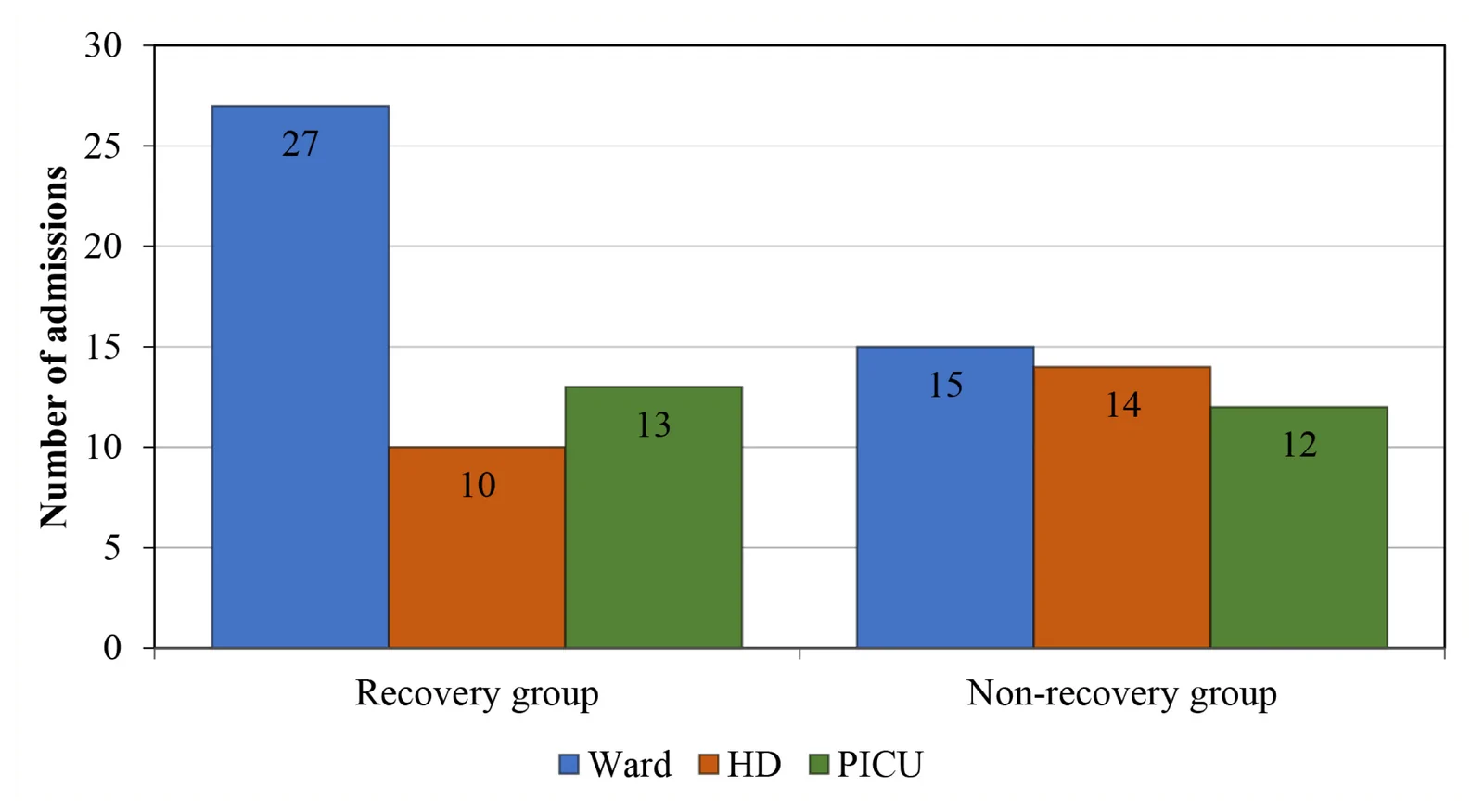
Frequency breakdown of patients admitted to various divisions depending on seizure duration (n = 91).

Patients were admitted to either the paediatric ward, HDU or ICU of SQUH. In total, 42 (46.67%) patients were admitted to the paediatric ward, 23 (25.56%) to the paediatric HDU, 24 (26.67%) to the paediatric ICU and 1 patient was admitted to both the paediatric HDU and ICU [Figure 1].

Out of the enrolled patients, 52 (55.9%) returned to baseline with good recovery, while 36 (38.7%) showed no recovery in terms of either disability or recurrence of convulsive status. The mortality rate was 5.38% (n = 5), while 2 (2.1%) cases were lost to follow-up. Neurological disability, according to the modified Rankin score, was mild in 11 (11.8%) cases, moderate in 12 (12.9%) cases and severe (with persistent vegetative state) in 7 (7.5%) cases.

Furthermore, aetiology was significantly associated with morbidity in terms of neurological disability and mortality (*P* = 0.021). The prolongation of CSE was associated with poorer outcomes (*P* = 0.041). Notably, there were no significant associations between age groups, seizure type, the timing of the first benzodiazepine injection and the patient outcomes.

## Discussion

The overall incidence of CSE is 20 cases per 100,000 children per year, with an associated mortality rate 3%. Consequently, CSE presents a continuous challenge for healthcare professionals in both pre-hospital and in-hospital settings.^19^ Given the high incidence of this common neurological emergency, CSE necessitates prompt identification and epidemiological surveillance to guide appropriate management. This study demonstrated that acute symptomatic seizures were the leading cause of CSE, followed by febrile status. These findings align with a study conducted in a developing country by Uzair *et al*., which reported a strong correlation between the cause of CSE and patient outcomes.^20^ Similarly, a study from the Kingdom of Saudi Arabia identified febrile seizure as the most common cause of CSE, followed by electrolyte imbalance and hydrocephalus.^21^

There is substantial data on the timing, choice and dosage of antiseizure medications for treating CSE. In the current study group, the most common first-line anticonvulsants were benzodiazepines, with diazepam used in 67.44% of cases and midazolam in 4.65%, reflecting the constantly available options in SQUH. This usage is consistent with findings from a similar study from Oman, where the same subgroups of benzodiazepines were used.^22^ Becker. noted that if intravenous (IV) access is available, IV lorazepam is at least as effective as, or even more effective than, IV diazepam or midazolam and has fewer side effects. In the absence of IV access, buccal and especially intramuscular midazolam can serve as effective first-line anticonvulsants in treating CSE in a hospital setting.^23^

In the current study, the most commonly used second-line antiseizure medications were phenytoin (42.86%) and levetiracetam (22.22%). This finding is consistent with a 2019 study that identified these drugs as the most effective second-line antiepileptics.^24^ Lyttle *et al*. also reported that levetiracetam has safety profiles and administration ease comparable to phenytoin, suggesting that the levetiracetam could be a suitable alternative as the first-choice, second-line anticonvulsant in the treatment of paediatric CSE.^24^

In this study, generalised seizures were the predominant seizure type (52.7%) associated with CSE and seizure semiology was significantly related to the aetiology of CSE (*P* ≤0.01), with acute aetiology being the most common. Acute aetiology typically involves diffuse cortical involvement, which may explain the high prevalence of generalised seizures observed in this study. In contrast, an international study conducted in Italy found focal convulsive seizures to be the most common semiology (50.8%), while generalised seizures were less common (32.3%) and nonconvulsive status (16.9%) was the least common semiology.^25^ Similar to the current study’s results, the Italian study found a statistically significant correlation between seizure semiology and aetiology (*P* ≤0.001).^25^ Additionally, the average age of patients in the present study was 4.84 ± 3.64 years, consistent with previous reports showing a higher prevalence of CSE in preschool children.^26^

Experts have hypothesised that younger children with CSE are more vulnerable to acute factors, such as febrile seizures, due to underdeveloped mechanisms of seizure control and the disruption of these mechanisms by minimal abnormalities in neuronal function. In this study, psychomotor development, which is closely related to nervous system maturity and dependent on genetic and structural factors, was normal in the majority of the children studied. To improve CSE prognosis and manage CSE effectively, it is crucial to understand the clinical profiles and factors that predict morbidity and mortality in children with CSE.

Richard’s study suggested that aetiology, rather than duration, is the primary determinant of outcomes for CSE patients, with those neurologically healthy before CSE having better outcomes.^27^ In contrast, the present study found that both aetiology and duration of CSE were significant predictors of outcome. The mortality rate in the current study was 5.38%, which is lower than the mortality rates reported by previous Indian studies (ranging from 14% to 33%). However, a recent study from a developing country reported a much higher mortality rate of 26.4%. The present study also identified longer duration of status, acute symptomatic aetiology and progressive encephalopathy as significant risk factors for mortality related to CSE.

Furthermore, Guterman and Vasquez reported that delayed or insufficient treatment with benzodiazepine can lead to lower efficacy, longer time to seizure cessation and an increased risk of refractoriness. These factors can result in more ICU admissions, as well as respiratory and hemodynamic complications, leading to worse outcomes.^29^^,^^30^ The present study observed a significant association between the time of the first benzodiazepine administration and CSE outcomes in terms of neurological disability and mortality. It noted that when IV benzodiazepine cannot be promptly administered, buccal or intramuscular midazolam should be considered.

### STRENGTHS AND LIMITATIONS

This study provides a comprehensive report of the varied aetiologies, management practices and acute outcomes, as well as outcome-predictive factors in children with CSE treated at a specialised centre in Oman. However, it is limited by being a single-centre retrospective study with a relatively small sample size. Additionally, it did not address other reported adverse outcomes of SE, such as cognitive and sensory impairments, hippocampal injury and the subsequent risk of epilepsy as long-term consequences.

## Conclusion

Acute symptomatic aetiology is the most common aetiology of CSE. Longer durations of CSE are associated with higher mortality and increased neurological disability in children. Prompt management of CSE and identification of the underlying cause can help reduce seizure duration and improve outcomes. Optimal care for children with CSE necessitates collaboration among community paediatricians, neurologists and emergency medical personnel.

## AVAILABILITY OF DATA

The data of this study can be provided upon reasonable request from the corresponding author.

## Figures and Tables

**Table 1:** Included patients’ characteristics, seizure type and aetiology of convulsive status epilepticus (N = 93)

Characteristic	n (%)
**Mean age in years ± SD**	4.84 ± 3.64
Gender	
Male	52 (55.9)
Female	41 (44.1)
Seizure type	
Generalised	49 (52.7)
Focal	17 (18.3)
Focal with bilateral tonic and clonic	24 (25.8)
Mixed (clonic, tonic, myoclonic)	3 (3.2)
Mean time between seizure onset and 1st BZD in minutes ± SD	25 ± 19
Aetiology	
Acute symptoms	35 (37.6)
Central nervous system infection	26 (27.9)
Acute demyelinating encephalomyelitis	7 (7.5)
Autoimmune encephalitis	2 (2.2)
Prolonged febrile seizure	29 (31.2)
Progressive encephalopathy	11 (11.8)
Neuro-degenerative disorder	2 (2.2)
Epileptic encephalopathy	7 (7.5)
Metabolic disorder	2 (2.2)
Static encephalopathy	4 (4.3)
Remote symptomatic	4 (4.3)
Structural epilepsy	3 (3.2)
Post meningitic sequelae	1 (1.1)
Idiopathic epilepsy	9 (9.7)
Unclassified	1 (1.1)

SD = standard deviation; BZD = benzodiazepine

**Table 2:** Frequency breakdown of use of antiseizure medication.

Medication Used	n (%)		
	1^st^ line	2^nd^ line	3^rd^ line
Diazepam	58 (67.44)	3 (4.76)	0 (0.00)
Phenytoin	9 (10.47)	27 (42.86)	10 (27.78)
Levetiracetam	4 (4.65)	14 (22.22)	6 (16.67)
Midazolam	4 (4.65)	9 (14.29)	11 (30.56)
Sodium valproate	6 (6.98)	2 (3.17)	3 (8.33)
Other medications	5 (5.82)	8 (12.70)	6 (16.67)

**Table 3:** Relationship of aetiology with age, seizure type and duration of status epilepticus (N = 93)

	n (%)							*P*-value
	Acute symptoms	Prolonged febrile seizure	Remote symptoms	Progressive encephalopathy	Static encephalopathy	Idiopathic epilepsy	Unclassified	
**Age in groups**								<0.01
1 month–2 years	18 (19.4)	17 (18.3)	1 (1.1)	2 (2.2)	1 (1.1)	2 (2.2)	0 (0.0)	
>2–6 years	9 (9.7)	12 (12.9)	1 (1.1)	3 (3.2)	3 (3.2)	6 (6.5)	1 (1.1)	
>6–12 years	8 (8.6)	0 (0.0)	2 (2.2)	6 (6.5)	0 (0.0)	1 (1.1)	0 (0.0)	
**Seizure type**								<0.01
Generalised	21 (22.6)	19 (20.4)	0 (0.0)	4 (4.3)	3 (3.2)	2 (2.2)	0 (0.0)	
Focal	3 (3.2)	10 (10.8)	1 (1.1)	3 (3.2)	0 (0.0)	0 (0.0)	0 (0.0)	
Mixed	0 (0.0)	0 (0.0)	0 (0.0)	2 (2.2)	1 (1.1)	0 (0.0)	0 (0.0)	
Focal with bilateral tonic and clonic	11 (11.9)	0 (0.0)	3 (3.2)	2 (2.1)	0 (0.0)	7 (7.5)	1 (1.1)	
**Duration of status epilepticus**								0.027
<1 hour	28 (30.1)	27 (29.0)	1 (1.1)	6 (6.5)	0 (0.0)	9 (9.7)	0 (0.0)	
1–6 hours	4 (4.3)	2 (2.2)	1 (1.1)	2 (2.2)	3 (3.2)	0 (0.0)	1 (1.1)	
>6 hours	3 (3.2)	0 (0.0)	2 (2.2)	3 (3.2)	1 (1.1)	0 (0.0)	0 (0.0)	

**Table 4:** Relationship between outcomes and predictive factors (N = 93)

Variable	n (%)			*P*-value
	Return to baseline (n = 52)	Neurological disability (n = 36)	Death (n = 5)	
**Aetiology**				0.021
Acute symptoms	15 (16.1)	18 (19.4)	2 (2.2)	
Prolonged febrile seizure	26 (28.0)	3 (3.2 )	0 (0.0)	
Remote symptoms	1 (1.1)	3 (3.2)	0 (0.0)	
Progressive encephalopathy	2 (2.2)	7 (7.5)	2 (2.2)	
Static encephalopathy	2 (2.1)	2 (2.2)	0 (0.0)	
Idiopathic epilepsy	6 (6.5)	3 (3.2)	0 (0.0)	
Unclassified	0 (0.0)	0 (0.0)	1 (1.1)	
**Duration of convulsive status epilepticus**				0.041
<1 hour	51 (54.8)	20 (21.5)	0 (0.0)	
1–6 hours	0 (0.0)	11 (11.8)	1 (1.1)	
>6 hours	1 (1.1)	5 (5.4)	4 (4.3)	
**Mean time between onset of seizures to 1st benzodiazepine injection**				<0.001
<10 minutes	11 (11.8)	7 (7.5)	0 (0.0)	
10–30 minutes	38 (40.9)	17 (18.3)	0 (0.0)	
>30 minutes	3 (3.2)	12 (13.0)	5 (5.4)	
